# Supplementary material for: Bayesian dynamic network modelling: an application to metabolic associations in cardiovascular diseases
Source: J Appl Stat. 2022 Sep 2;51(1):114–38. doi: 10.1080/02664763.2022.2116746 (PMC10763914; doi:10.1080/02664763.2022.2116746)
Supplement: Supplemental Material [file CJAS_A_2116746_SM2225.pdf]

RESEARCH ARTICLE

Supplemental Material to 'Bayesian Dynamic Network Modelling: an application to metabolic associations in cardiovascular diseases'

Marco Molinari[a], Andrea Cremaschi [b], Maria De Iorio [a,b,c], Nishi Chaturvedi[d], Alun Hughes[d] and Therese Tillin[d]

[a]Department of Statistical Science, University College London, UK; [b]Singapore Institute for Clinical Sciences, A*STAR, Singapore;[c]Yong Loo Lin School of Medicine, National University of Singapore, Singapore;[d]Department of Population Science and Experimental Medicine, University College London, UK.

ARTICLE HISTORY
Compiled July 29, 2022

ABSTRACT
This document contains additional information relative to the manuscript 'Bayesian Dynamic Network Modelling: an application to metabolic associations in cardiovascular diseases' and it is organised as follows. Section 1 contains the details of the MCMC algorithm needed to perform posterior inference. We provide both the `Stan` code and the Gibbs sampling scheme. Section 2 shows additional figures and tables referenced in the main text.

KEYWORDS
Dynamic Shrinkage Priors; Gibbs Sampling; Graphical Models; Metabolomics; Nodewise Regression

CONTACT Maria De Iorio. Email: m.deiorio@ucl.ac.uk

1. MCMC Algorithm

Here we provide the `Stan` code and the details of the Gibbs sampling algorithm to perform posterior inference of the multiple groups dynamic nodewise regression model.

Stan *code*

```
data {
int < lower = 1 > NT;             // Time points
int < lower = 1 > n_t[ NT ]; // Number of observations for each time point
int < lower = 1 > Ngr; // Number of groups
int < lower = 1 > n_groups[ Ngr*NT ]; // Dimension of the groups for each time
int < lower = 1 > m;                 // Number of regressors
int < lower = 1, upper = Ngr > G_t[ sum(n_groups) ];    // Vector with groups membership
vector [ sum(n_t) ] y_t;             // Response variable
row_vector[m] X_t[ sum(n_t) ];         // Input matrix (= Y_-j)
// ---------------
real < lower = 0 > scale_global_tau_0[ Ngr ]; // prior scale for the global shrinkage parameter Tau
real < lower = 1 > df_global_0;  // degrees of freedom for Tau
real < lower = 1 > df_global_j;
real < lower = 1 > df_local_t; // degrees of freedom for Lambdas
matrix <lower = 0> [m, Ngr] devs_X_t[ NT ]; // Deviances of X (diag of XtX )
// ------ Params for the regularisation of the Horseshoe
real <lower = 0> slab_scale_c_t;  // Slab of student-t
real <lower = 0> slab_df_c_t;  // df of student-t
// Parameters Beta on Phi_j
real <lower = 0> phi_ab[ 2 ];
real <lower = 0> sigma_prior;
}
transformed data{
int n_cumsum_gr[ Ngr * NT ]; // Vector of cumulative sum of observations in groups per time
int pos;
pos = 1;
for(tt in 1:NT){
for(gr in 1:Ngr){
if( pos == 1) n_cumsum_gr[ pos ] = 1;
if( pos > 1) n_cumsum_gr[ pos ] = n_cumsum_gr[ pos - 1 ] + n_groups[ pos - 1 ];
pos += 1;
}
}
}
parameters {
vector < lower = 0 > [ Ngr * NT ] sigma_t; // Time varying noise std
// Parameters T
vector[ m * Ngr ] z_t[ NT ]; //Innovations for Beta_t
// Tau_j
vector < lower = 0 > [ m * Ngr ] aux1_global_j;
vector < lower = 0 > [ m * Ngr ] aux2_global_j;
// Tau_0
real < lower = 0 > aux1_global_0;
real < lower = 0 > aux2_global_0;
// Local Lambda_jt
vector < lower = 0 > [ m * Ngr ] aux1_local_t[ NT ];
vector < lower = 0 > [ m * Ngr ] aux2_local_t[ NT ];
vector < lower = 0 > [ Ngr ] aux_c_t[ NT ];
// Autoregressive Parameter phi_j for the dynamic HS
vector < lower = 0 , upper = 1 > [ m * Ngr ] phi_j_pos;
}
transformed parameters {
vector < lower = -1 , upper = 1 > [ m * Ngr ] phi_j;
// Tau_j
```

```
vector < lower = 0 > [ m ] tau_j[ Ngr ];
// Tau_0
real < lower = 0 > tau_0[ Ngr ];
// Local Lambda_j,t
vector < lower = 0 > [ m ] lambda_t[ NT * Ngr ];
vector < lower = 0 > [ m ] lambda_tilde_t[ NT * Ngr ]; // truncated local shrinkage parameter
vector < lower = 0 > [ Ngr ] c_t[ NT ]; // c for reg HS
// log_scale variance h_j,t
vector [ m ] log_h[ NT * Ngr ];
vector < lower = 0 > [ m ] exp_h[ NT * Ngr ];
// Beta_t
vector[ m ] beta_t[ NT * Ngr ];
vector [ sum( n_t ) ] f_t;
vector < lower = 0 > [ sum(n_t) ] sigma_long;  // To store the sigma for each obs
// <<<<<<<<<<<<<<<<<<<<<<>>>>>>>>>>>>>>>>>>>>>>>>
// <<<<<<<<<<<<<<< Now operations >>>>>>>>>>>>>>
// <<<<<<<<<<<<<<<<<<<<<<>>>>>>>>>>>>>>>>>>>>>>>>
// Adjust phi_j
phi_j = (phi_j_pos * 2) - 1;
// In Time == 1
c_t[ 1 ] = slab_scale_c_t * sqrt( aux_c_t[ 1 ] );
for(gr in 1:Ngr){
// Global Shrinkage parameters
tau_j[ gr ] = aux1_global_j[((gr-1)*m + 1):((gr-1)*m + m)] .*
sqrt( aux2_global_j[((gr-1)*m + 1):((gr-1)*m + m)] );
tau_0[ gr ] = aux1_global_0 * sqrt( aux2_global_0 ) * scale_global_tau_0[ gr ] * sigma_t[ gr ];
// tau_j[ gr ] = aux1_global_j .* sqrt( aux2_global_j[ ((gr-1)*m + 1):((gr-1)*m + m) ] );
lambda_t[ gr ] = aux1_local_t[ 1 ][((gr-1)*m + 1):((gr-1)*m + m)].*
sqrt( aux2_local_t[ 1 ][((gr-1)*m + 1):((gr-1)*m + m)] );
lambda_tilde_t[ gr ] = sqrt( ( c_t[ 1 ][ gr ]^2 *
square( lambda_t[ gr ] ) ) ./ ( c_t[ 1 ][ gr ]^2 +
square( tau_j[ gr ] ) .* square( lambda_t[ gr ] )));
// Begin the creation og log-volatility h
log_h[ gr ] = 2*log( lambda_tilde_t[ gr ] ) + 2*( log( tau_j[ gr ] ) + log(tau_0[ gr ]) );
exp_h[ gr ] = exp( 0.5 * log_h[ gr ] );
}
// For time > 1
for(tt in 2:NT){
c_t[ tt ] = slab_scale_c_t * sqrt( aux_c_t[ tt ] );
for(gr in 1:Ngr ){
lambda_t[ (tt-1)*Ngr + gr ] = aux1_local_t[ tt ][((gr-1)*m + 1):((gr-1)*m + m)] .*
sqrt( aux2_local_t[ tt ][((gr-1)*m + 1):((gr-1)*m + m)] );
lambda_tilde_t[ (tt-1)*Ngr + gr ] = sqrt( ( c_t[ tt ][ gr ]^2 *
square( lambda_t[ (tt-1)*Ngr + gr ] ) ) ./
( c_t[ tt ][ gr ]^2 + square( tau_j[ gr ] ) .* square(lambda_t[ (tt-1)*Ngr + gr ])));
for(jj in 1:m){
log_h[ (tt-1)*Ngr + gr ][jj] = 2*log( lambda_tilde_t[ (tt-1)*Ngr + gr ][ jj ] ) +
2*( log( tau_j[ gr ][jj]) + log( tau_0[ gr ] ) ) +
phi_j[ (gr-1)*m + jj ] * ( log_h[ (tt-2)*Ngr + gr ][jj] -
2*( log( tau_j[ gr ][jj]) + log( tau_0[ gr ] )));
exp_h[ (tt-1)*Ngr + gr ][jj] = exp( 0.5 * log_h[ (tt-1)*Ngr + gr ][jj] );
}
}// gr
}// tt
// Beta_t
for(gr in 1:Ngr){
beta_t[ gr ] = z_t[ 1 ][((gr-1)*m + 1):((gr-1)*m + m)] .* exp_h[ gr ];
}
for(tt in 2:NT){
for(gr in 1:Ngr){
beta_t[ (tt-1)*Ngr + gr ] = beta_t[ (tt-2)*Ngr + gr ] + z_t[ tt ][((gr-1)*m + 1):((gr-1)*m + m)] .*
exp_h[ (tt-1)*Ngr + gr ];
}
}
```

```
// Mean function
{
int ii_ind;
ii_ind = 1;
for(tt in 1:NT ){
for(gr in 1:Ngr){
for(ii in 1:n_groups[ (tt-1)*Ngr + gr ] ){
sigma_long[ ii_ind ] = sigma_t[ (tt-1)*Ngr + gr ];
f_t[ ii_ind ] = X_t[ ii_ind ] * beta_t[ (tt-1)*Ngr + gr ];
ii_ind += 1;
}
}
}
}
}
model {
// Here we use auxiliary variables for tau and lambda
// Locals shared across groups
sigma_t ~ inv_gamma( 0.5, 0.5 );
phi_j_pos ~ beta( phi_ab[1], phi_ab[2] );
// One global tau for each group
aux1_global_0 ~ std_normal();
aux2_global_0 ~ inv_gamma( 0.5 * df_global_0, 0.5 * df_global_0 );
aux1_global_j ~ std_normal();
aux2_global_j ~ inv_gamma( 0.5 * df_global_j, 0.5 * df_global_j );
for(tt in 1:NT){
aux2_local_t[ tt ] ~ inv_gamma( 0.5 * df_local_t, 0.5 * df_local_t );
aux1_local_t[ tt ] ~ std_normal();
aux_c_t[ tt ] ~ inv_gamma( 0.5 * slab_df_c_t, 0.5 * slab_df_c_t );
z_t[ tt ] ~ std_normal();
}
// Likelihood
y_t ~ normal(f_t , sigma_long );
}
generated quantities {
// Vector of k_j, the pseudo inclusion probability
vector [ m ] k_j_t[ NT * Ngr ];
// Vector to hold precision Omega elements for the current equation
vector [ m ] omega_vec_t[ NT * Ngr ];
// Elements of Omega
for(tt in 1:NT){
for(gr in 1:Ngr ){
for(jj in 1:m){
k_j_t[ (tt-1)*Ngr + gr ][jj] = 1/( 1 + 1/square( sigma_t[ gr ] ) * (exp_h[ (tt-1)*Ngr + gr ][jj] *
exp_h[ (tt-1)*Ngr + gr ][jj]) * devs_X_t[ tt ][ jj, gr ] );
omega_vec_t[ (tt-1)*Ngr + gr ][ jj ] = (- beta_t[ (tt-1)*Ngr + gr ][ jj ] ) / square( sigma_t[ gr ] );
}
}
}
}
```

Gibbs sampling algorithm

Here we provide details of the Gibbs sampling. Our starting point is the algorithm described in [41] and we extend it to allow for multiple groups of different sample sizes. The sampler consists of two main components: a stochastic volatility sampling algorithm [40] augmented with a Polya-Gamma sampler [58], and a Cholesky Factor Algorithm [62] to sample the regression coefficients in the dynamic linear model. We provide details of the update steps that differ from the original paper, for the others refer to [41].

- *Resampling the log-variances h_{jrt} given the rest.* [55] propose a method to sample directly from the full-conditional distribution of $\boldsymbol{h_{jr}} = (h_{jr1}, \ldots, h_{jrT})$, on the log-scale, where the ensuing log-chi-square distribution of the likelihood $\log(\gamma^2_{jrt})$, from

$$\log\left((\beta_{jrt} - \beta_{jrt-1})^2\right) = \log(\gamma^2_{jrt}) + h_{jrt}$$

  is approximated via a known normal mixture approximation of 10 components. To sample the new log-variances we use the all-without-a-loop (AWOL) sampler of [40], which allows to sample $\boldsymbol{h_{jr}}$ jointly without the need of a sequential algorithm. Conditional on all the other parameters the log-variances are independent across groups. Resampling proceeds as in [41].
- *Resampling the mixture components indicator s_{jrt} given the rest.* The discrete mixture probabilities are straightforward to update. The prior mixture probabilities are the pre-specified mixing proportions given by [55] and the likelihood is $\log(\gamma^2_{jrt} + c) \sim \mathrm{N}(h_{jrt} + m_{s_{jrt}}, v_{s_{jrt}})$, where c is a small offset to avoid numerical underflows and m and v are the pre-specified mean and variance, respectively, of the 10 mixture components. Resampling proceeds as in [41], independently for each group.
- *Resampling the mean parameters μ_{jr} given the rest.* The update of μ_{jr} is done independently for each group. We can re-write the log-variance equation in (8) as an ordinary linear regression, where the parameters μ_{jr} play the role of regression coefficients, as follows:

$$\begin{aligned} h_{jrt} &= \mu_{jr} + \phi_{jr}(h_{jrt-1} - \mu_{jr}) + \eta_{jrt} \\ \tilde{h}_{jrt} &= \mu_{jr}(1 - \phi_{jr}) + \eta_{jrt} \end{aligned}$$

  where $\tilde{h}_{jrt} = h_{jrt} - \phi_{jr} h_{jrt-1}$ and $\tilde{h}_{jrt} \sim \mathrm{N}\left(\mu_{jr}(1 - \phi_{jr}), \xi^{-1}_{\eta_{jrt}}\right)$. Finally, we can write

$$\tilde{\tilde{h}}_{jrt} \sim \mathrm{N}\left(\mu_{jr} z_{jrt}, 1\right)$$

  where $z_{jrt} = (1 - \phi_{jr})\sqrt{\xi_{\eta_{jrt}}}$ are the regression covariates, μ_{jr} are the regression coefficient and $\tilde{\tilde{h}}_{jrt} = \tilde{h}_{jrt}\sqrt{\xi_{\eta_{jrt}}}$, for $t = 2, \cdots, T$, are the response variables. The posterior follows from the standard update of a Normal prior with Normal likelihood. For $j = 1, \ldots, p$ and $r = 1, \ldots, R$ we sample from

$$\begin{aligned} \mu_{jr} \mid rest &\sim \mathrm{N}\left(l_\mu / q_\mu, 1/q_\mu\right) \\ q_\mu &= \xi_{\mu_{jr}} + \xi_{\eta_{jr1}} + \sum_{t=2}^{T} z^2_{jrt} \\ l_\mu &= \xi_{\mu_{jr}} \mu_0 + h_{jr1} \xi_{\eta_{jr1}} + \sum_{t=2}^{T} \tilde{\tilde{h}}_{jrt} z_{jrt} \end{aligned}$$

- *Resampling the mean parameter μ_0 given the rest.* The posterior distribution of μ_0 follows from the standard update of a Normal prior and Normal likelihood

represented by μ_{jr}. We sample from

$$\mu_0 \mid rest \sim \mathrm{N}(l_0/q_0, 1/q_0)$$
$$q_0 = \xi_{\mu_0} + \sum_{r=1}^{R}\sum_{j=1}^{p} \xi_{\mu_{jr}}$$
$$l_0 = \sum_{r=1}^{R}\sum_{j=1}^{p} \xi_{\mu_{jr}}\mu_{jr}$$

- *Resampling the autoregressive coefficients ϕ_{jr} given the rest.* The new value of ϕ_{jr} is drawn via slice sampler, independently for each group. The parametrisation $(\phi_{jr}+1)/2 \sim \mathrm{Beta}(a_\phi, b_\phi)$ implies that $|\phi_{jr}| < 1$, which ensures a stationary stochastic process for $\boldsymbol{h_{jr}}$. Resampling is performed as in [41].
- *Resampling the Polya-Gamma mixing parameters $\xi_{\eta_{jrt}}$, $\xi_{\mu_{jr}}$ and ξ_{μ_0} given the rest.* This step is a conjugate update of a Polya-Gamma prior given a Gaussian likelihood [58].

$$\xi_{\eta_{jrt}} \mid rest \sim \text{Polya-Gamma}(1, \eta_{jrt}), \qquad \forall j, r, t$$
$$\xi_{\mu_{jr}} \mid rest \sim \text{Polya-Gamma}(1, \mu_{jr} - \mu_{0r}), \qquad \forall j, r$$
$$\xi_{\mu_0} \mid rest \sim \text{Polya-Gamma}\,(1, \mu_0)$$

- *Resampling the regression coefficients $\boldsymbol{\beta}_{rt}$ given the rest.* Conditional on the rest, the regression coefficients of each group are independent. We perform a joint update of $\boldsymbol{\beta} = (\boldsymbol{\beta}_1^T, \dots, \boldsymbol{\beta}_t^T, \dots, \boldsymbol{\beta}_T^T)$, where $\boldsymbol{\beta}_t = (\beta_{1t}, \dots, \beta_{pt})$, exploiting the block-diagonal structure of the posterior precision matrix of $\boldsymbol{\beta}$. The posterior distribution is (omitting the group subscript for ease of notation)

$$\boldsymbol{\beta} \mid rest \sim \mathrm{N}\left(Q_\beta^{-1}\boldsymbol{m}_\beta, Q_\beta^{-1}\right)$$
$$Q_\beta = A_\sigma + A_h$$

where A_σ is a $Tp \times Tp$ block-diagonal matrix defined as follows:

$$A_\sigma = \begin{bmatrix} X_1^T X_1/\sigma_1^2 & 0 & \cdots & \cdots & 0 \\ \vdots & \ddots & \vdots & \vdots & \vdots \\ 0 & \cdots & X_t^T X_t/\sigma_t^2 & \cdots & 0 \\ \vdots & \vdots & \vdots & \ddots & \vdots \\ 0 & 0 & \cdots & \cdots & X_T^T X_T/\sigma_T^2 \end{bmatrix}$$

and A_h is a $Tp \times Tp$ matrix defined as follows:

$$A_h = \left(D^T \otimes I_p\right)\Sigma_h^{-1}\left(D \otimes I_p\right)$$

where $\otimes$ denotes the Kronecker product, D is a $T \times T$ tri-diagonal matrix with diagonal entries equal to 1 and first off-diagonal elements equal to -1, I_p is the $p \times p$ identity matrix and Σ_h^{-1} is a $Tp \times Tp$ diagonal matrix with diagonal entries

Setting		HMC	Gibbs
	$M = 20$	0.26	0.28
$T = 3$	$M = 50$	1.18	1.11
	$M = 100$	2.36	4.00
	$M = 20$	1.68	0.34
$T = 10$	$M = 50$	9.75	1.60
	$M = 100$	75.10	7.27

Table 1. Computational times (seconds per iteration) for the HMC algorithm as implemented in `Stan` and the blocked Gibbs sampling MCMC scheme as implemented in `R`. Simulations are run for different values of the number M of nodes and number T of time points, with $N = 100$ and $R = 2$. Computational times are recorded on a Linux machine with Intel Core i7 1.8 GHZ.

equal to

$$(\exp(h_{11}/2), \dots, \exp(h_{p1}/2), \dots, \exp(h_{1T}/2), \dots, \exp(h_{pT}/2))$$

The posterior mean $\boldsymbol{m}_\beta$ is a Tp-dimensional vector defined as:

$$\boldsymbol{m}_\beta = \left(X_1^T \boldsymbol{y}_1/\sigma_1^2, \dots, X_t^T \boldsymbol{y}_t/\sigma_t^2, \dots, X_T^T \boldsymbol{y}_T/\sigma_T^2\right)$$

- *Resampling the observation error variances σ_{rt}^2 given the rest.* This step is a conjugate update of an Inverse-Gamma prior given a Gaussian likelihood. For each group r and time t we sample:

$$\sigma_{rt}^2 \mid rest \sim \text{Inverse-Gamma}\left(a_\sigma + \frac{n_{rt}}{2}, b_\sigma + \frac{\sum_{i=1}^{n_{rt}}(y_{irt} - \boldsymbol{x}_{irt}\boldsymbol{\beta}_{rt})^2}{2}\right)$$

2. Additional Figures and Tables

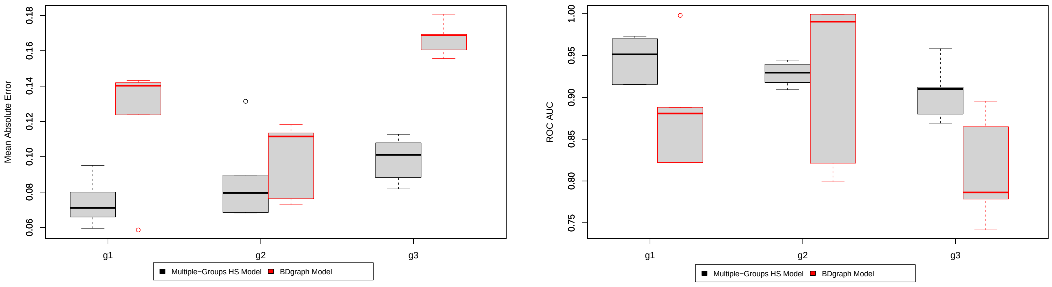

Figure 1. First simulation scenario. Mean Absolute Error (left panel) and AUC (right panel) for the comparison between the multiple groups nodewise regression model in (7) and the package `BDgraph`. The boxplots of the MAE and AUC are obtained over twenty replicates for each model and by adopting the *AND* rule. The MAE and the AUC are compared within each of the three groups G_1, G_2 and G_3. The nodewise model works better both in terms of MAE, indicating a more precise estimation of Ω, and in terms of AUC, denoting a better recovery of the graph structure within each group.

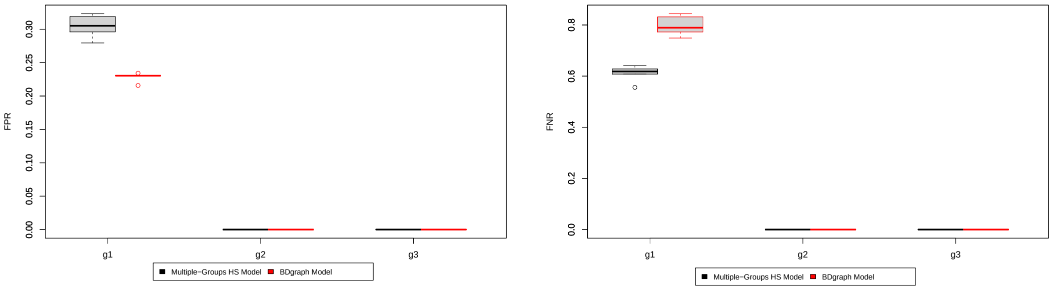

Figure 2. First simulation scenario. False positive rates (FPR, left panel) and false negative rates (FNR, right panel) for the comparison between the multiple groups nodewise regression model in (7) and the package `BDgraph`. The boxplots of the FPR and FNR are obtained over twenty replicates for each model and by adopting the *AND* rule. The FPR and the FNR are compared within each of the three groups G_1, G_2 and G_3. The FPR and the FNR are comparable in both models, and in particular equal to zero for groups G_2 and G_3.

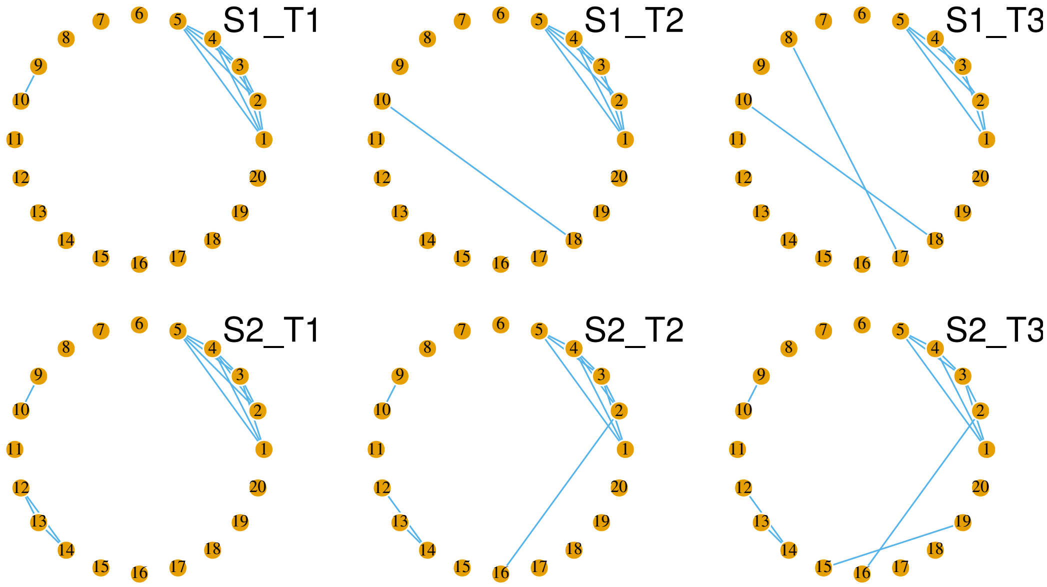

Figure 3. Networks generated in the second simulation scenario and used to simulate the dataset within each group and evolving over time.

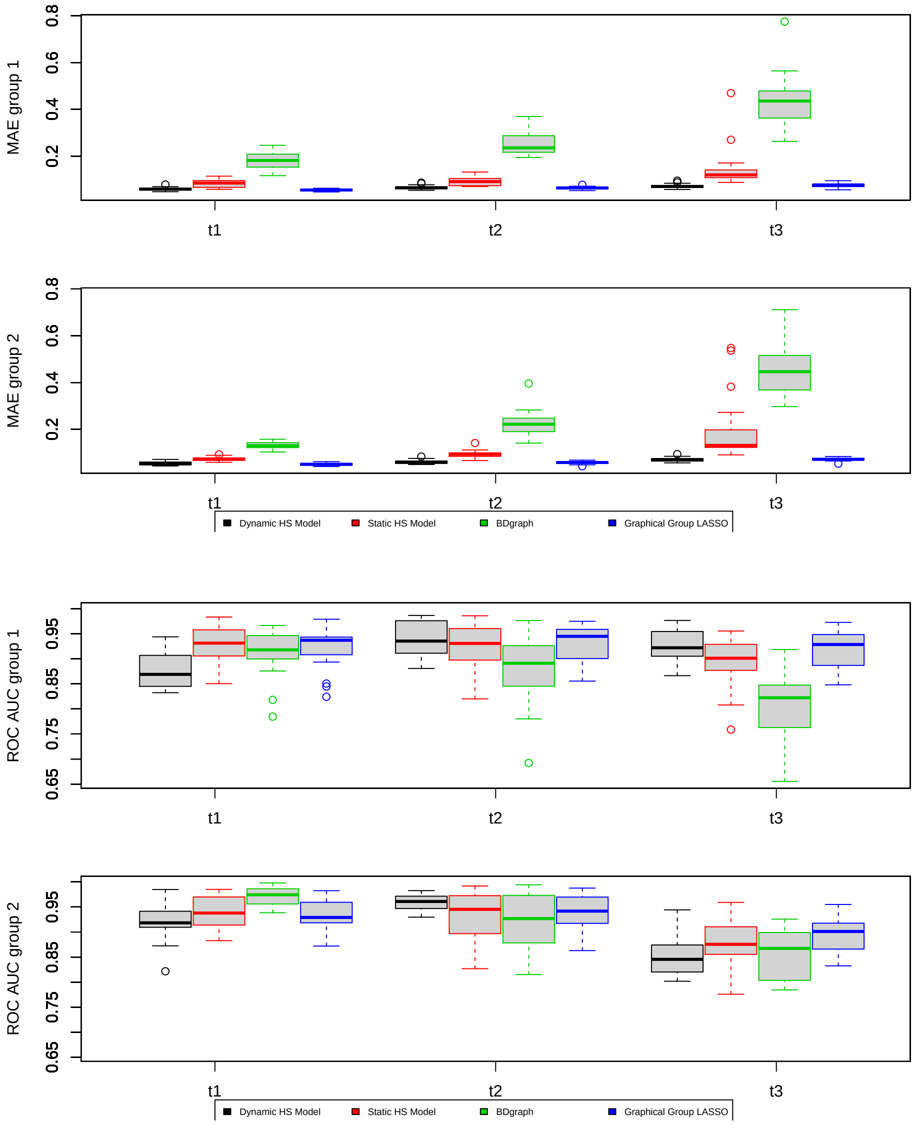

Figure 4. Second simulation scenario. Mean Absolute Error (top panel) and AUC (bottom panel) comparison between the dynamic model in (9) (black), the static model in (7) (red), `BDgraph` (green) and Graphical Group LASSO (blue). The boxplots of the MAE and AUC are obtained over twenty replicates for each model and by adopting the *AND* rule. Each row in the Figure refers to a group, and the MAE and the AUC are compared at each time point. The MAE indicates that the proposed model performs well in the estimation of the precision matrix. In terms of AUC and graph recovery, the dynamic HS model yields better estimates in two of the time points for the first group, and in the second time point for the second group. In this simulation settings, no method consistently outperforms the others.

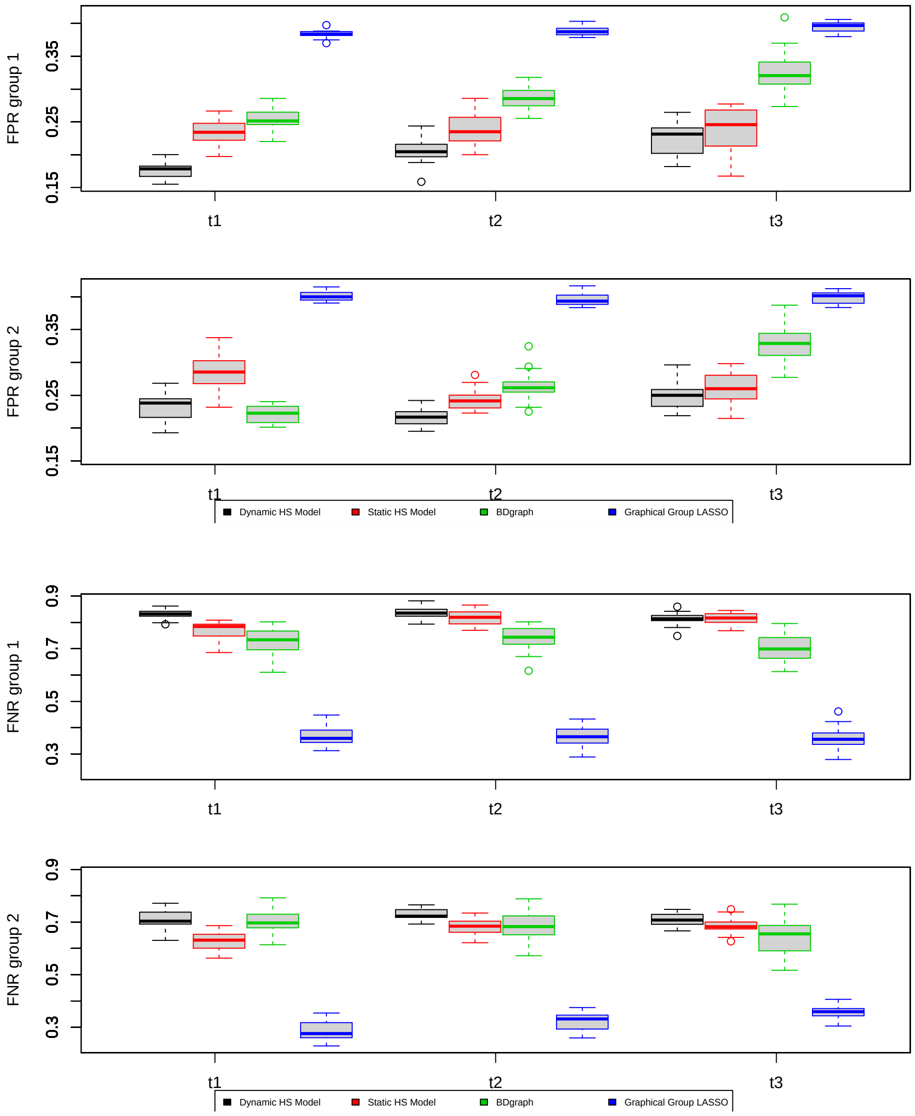

Figure 5. Second simulation scenario. False positive rates (FPR) and false negative rates (FNR) for the dynamic model in (9) (black), the static model in (7) (red), `BDgraph` (green) and Graphical Group LASSO (blue). The boxplots are obtained over twenty replicates for each model and by adopting the *AND* rule. Each row in the Figure refers to a group, and the FPR and FNR are compared at each time point. The results obtained with the proposed dynamic and static models are comparable at most of time points and in both groups. Similar results are observed for the BDgraph model. Graphical Group LASSO presents differs the most from the other models.

Table 2. Metabolites extended names. Each lipoprotein is divided into triglycerides, cholesteryl esters, free cholesterol and phospholipids.

Abbreviation	Full name	Differentially expressed
acace	Acetoacetate	
ace	Acetate	
ala	Alanine	
alb	Albumin	
apoa1	Apolipoprotein A-I	
apob	Apolipoprotein B	✓
bohbut	3-hydroxybutyrate	
ce	Cholesteryl Esters	✓
cit	Citrate	
crea	Creatinine	
dha	22:6, docosahexaenoic acid	
faw3	Omega-3 fatty acids	
faw6	Omega-6 fatty acids	✓
fc	Free Cholesterol	✓
glc	Glucose	✓
gln	Glutamine	✓
glol	Glycerol	
gly	Glycine	
gp	Glycoprotein acetyls, mainly a1-acid glycoprotein	✓
his	Histidine	
ile	Isoleucine	
la	18:2, linoleic acid	✓
lac	Lactate	✓
leu	Leucine	
mufa	Monounsaturated fatty acids; 16:1, 18:1	✓
pc	Phosphatidylcholines and other cholines	
phe	Phenylalanine	
pl	Phospholipids	✓
pufa	Polyunsaturated fatty acids	
pyr	Pyruvate	
sfa	Saturated fatty acids	
sm	Sphingomyelins	✓
tg	Triglycerides	✓
tyr	Tyrosine	
unsatdeg	Estimated degree of unsaturation	
val	Valine	
lipids_s_hdl	Lipids compounds in small HDL	
lipids_m_hdl	Lipids compounds in medium HDL	
lipids_l_hdl	Lipids compounds in large HDL	
lipids_xl_hdl	Lipids compounds in extra large HDL	
lipids_s_ldl	Lipids compounds in small LDL	
lipids_m_ldl	Lipids compounds in medium LDL	
lipids_l_ldl	Lipids compounds in large LDL	
lipids_idl	Lipids compounds in IDL	
lipids_xs_vldl	Lipids compounds in extra small VLDL	
lipids_s_vldl	Lipids compounds in small VLDL	
lipids_m_vldl	Lipids compounds in medium VLDL	
lipids_l_vldl	Lipids compounds in large VLDL	
lipids_xl_vldl	Lipids compounds in extra large VLDL	
lipids_xxl_vldl	Lipids compounds in extra extra large VLDL	

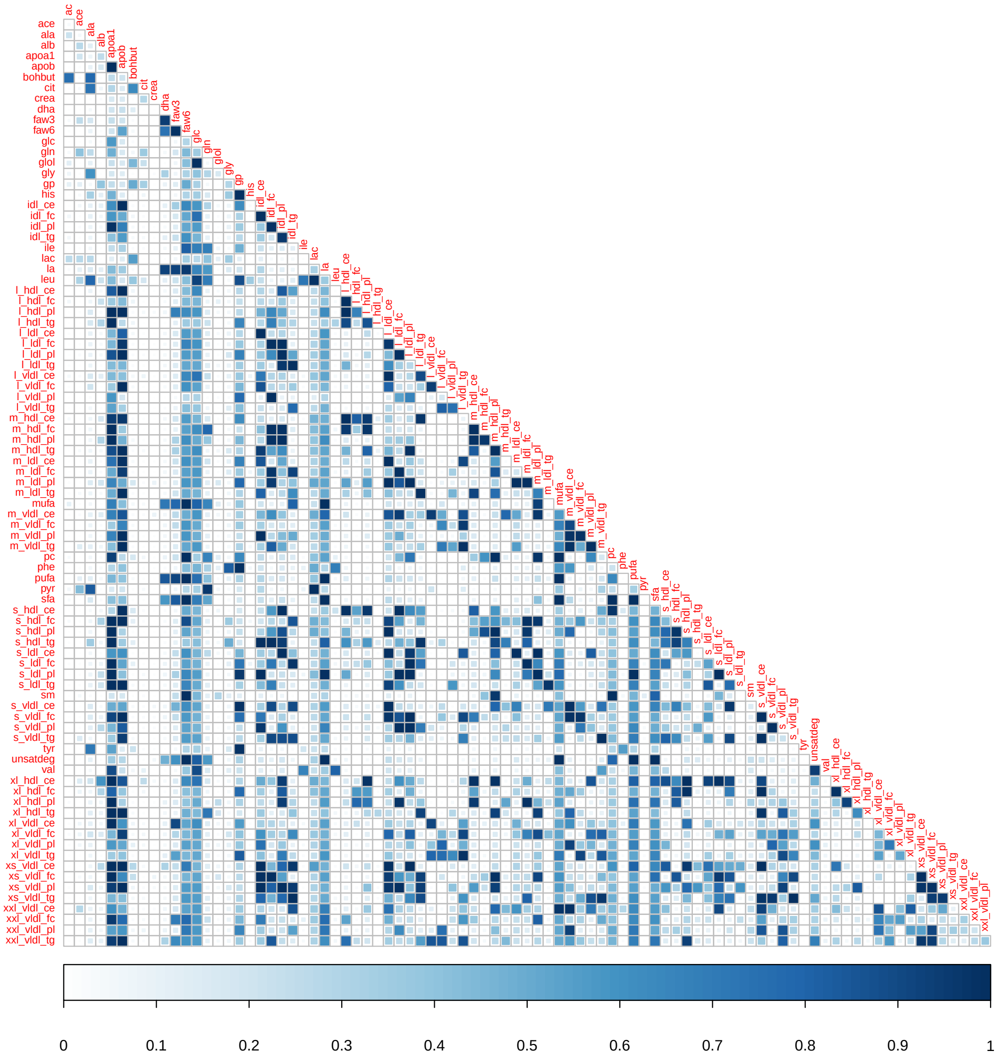

Figure 6. Posterior mean of the pseudo inclusion probability parameters κ_{jl} (see Eq. (5)) for the European ethnicity at baseline. The edges in the individual networks are selected when the posterior mean of $(1 - \kappa_{jl})$ is greater than 0.5.

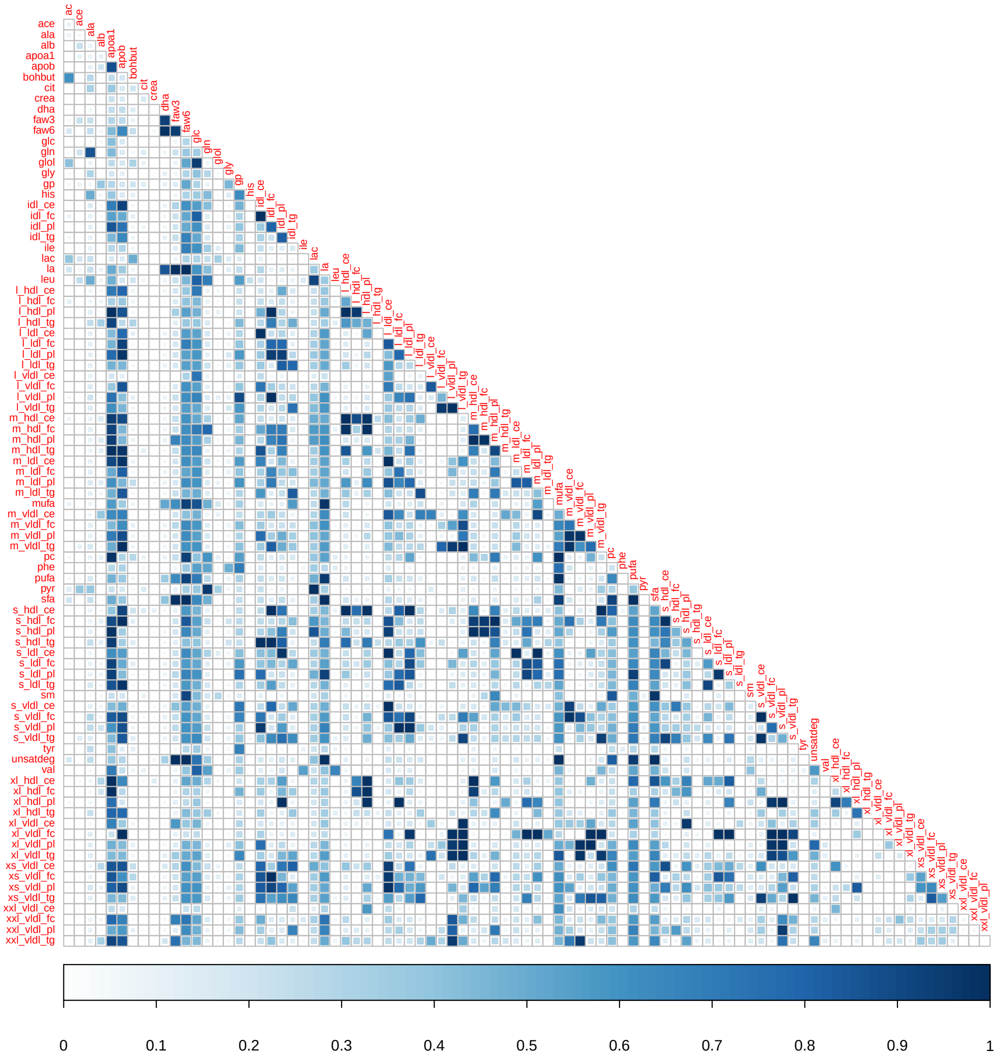

Figure 7. Posterior mean of the pseudo inclusion probability parameters κ_{jl} (see Eq. (5)) for the European ethnicity at follow-up. The edges in the individual networks are selected when the posterior mean of $(1 - \kappa_{jl})$ is greater than 0.5.

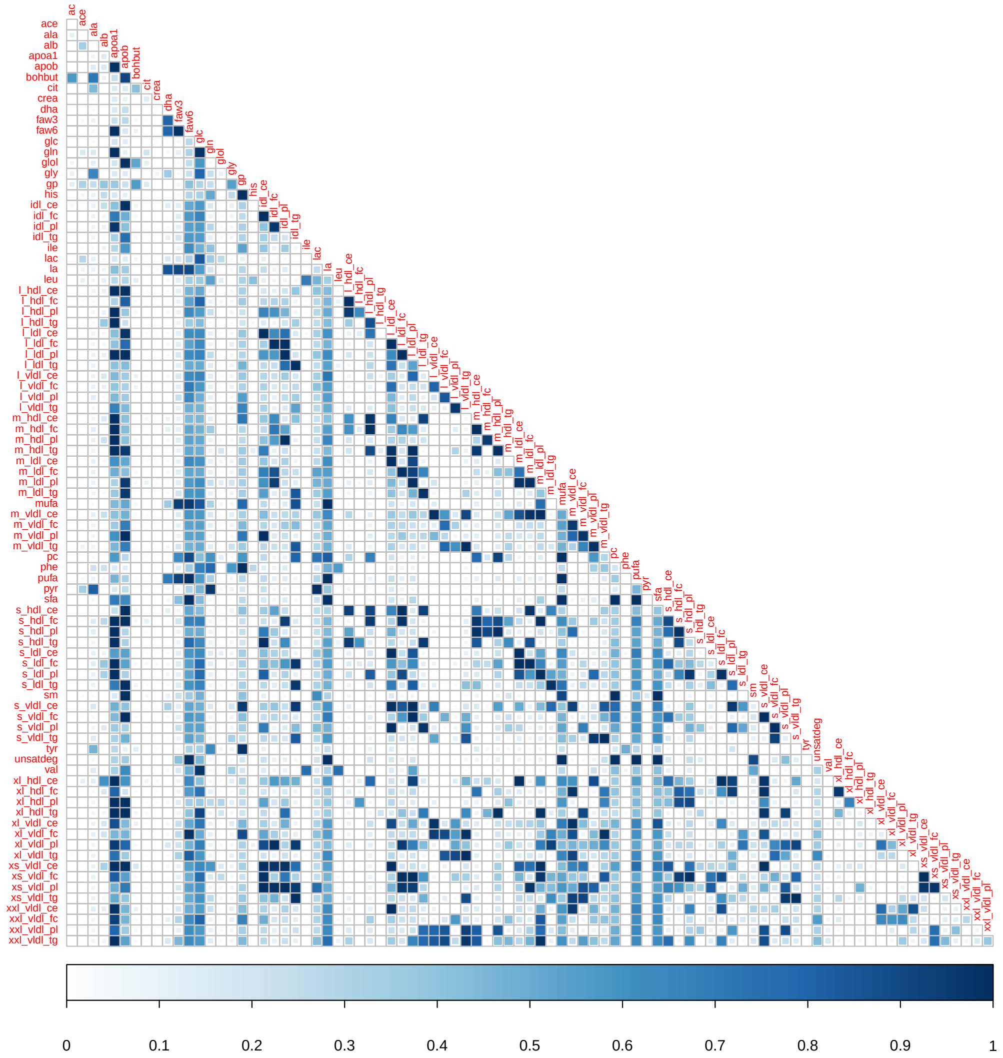

Figure 8. Posterior mean of the pseudo inclusion probability parameters κ_{jl} (see Eq. (5)) for the South-Asian ethnicity at baseline. The edges in the individual networks are selected when the posterior mean of $(1 - \kappa_{jl})$ is greater than 0.5.

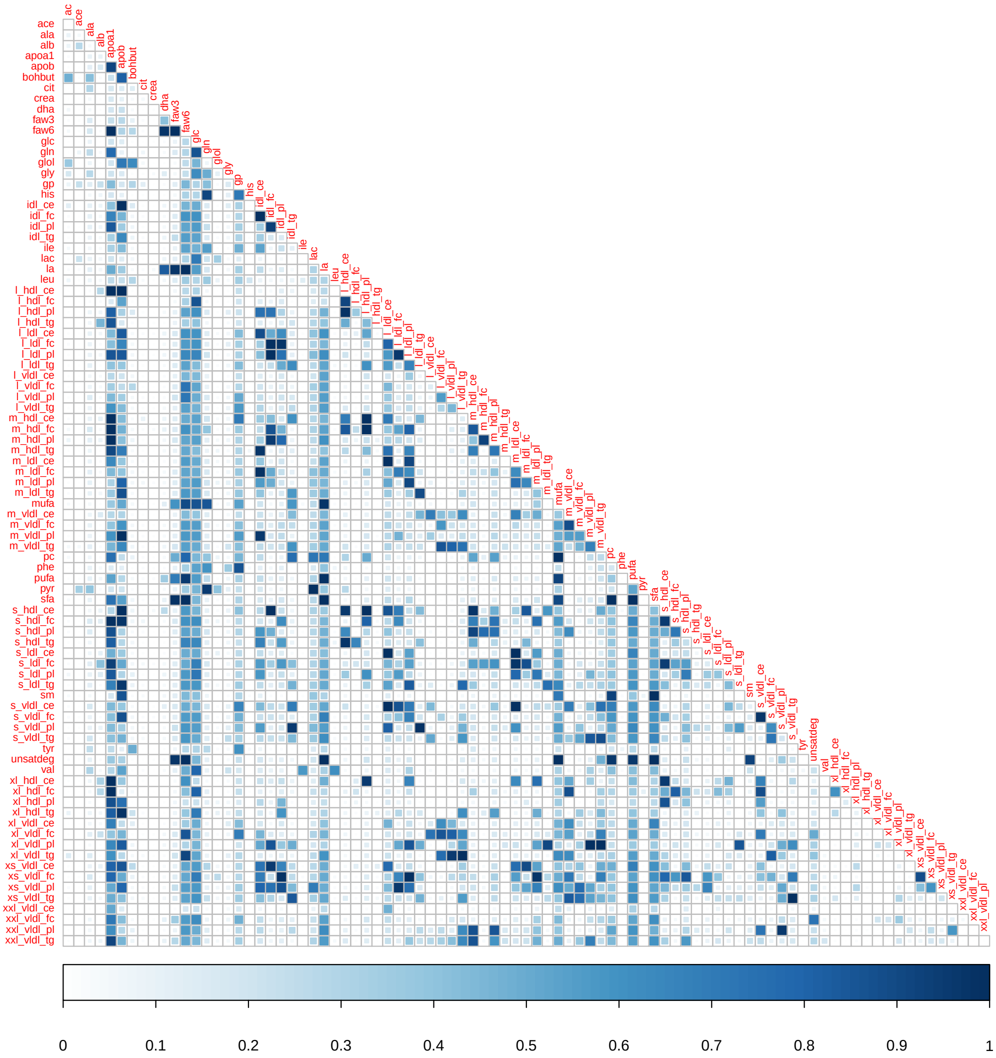

Figure 9. Posterior mean of the pseudo inclusion probability parameters κ_{jl} (see Eq. (5)) for the South-Asian ethnicity at follow-up. The edges in the individual networks are selected when the posterior mean of $(1-\kappa_{jl})$ is greater than 0.5.

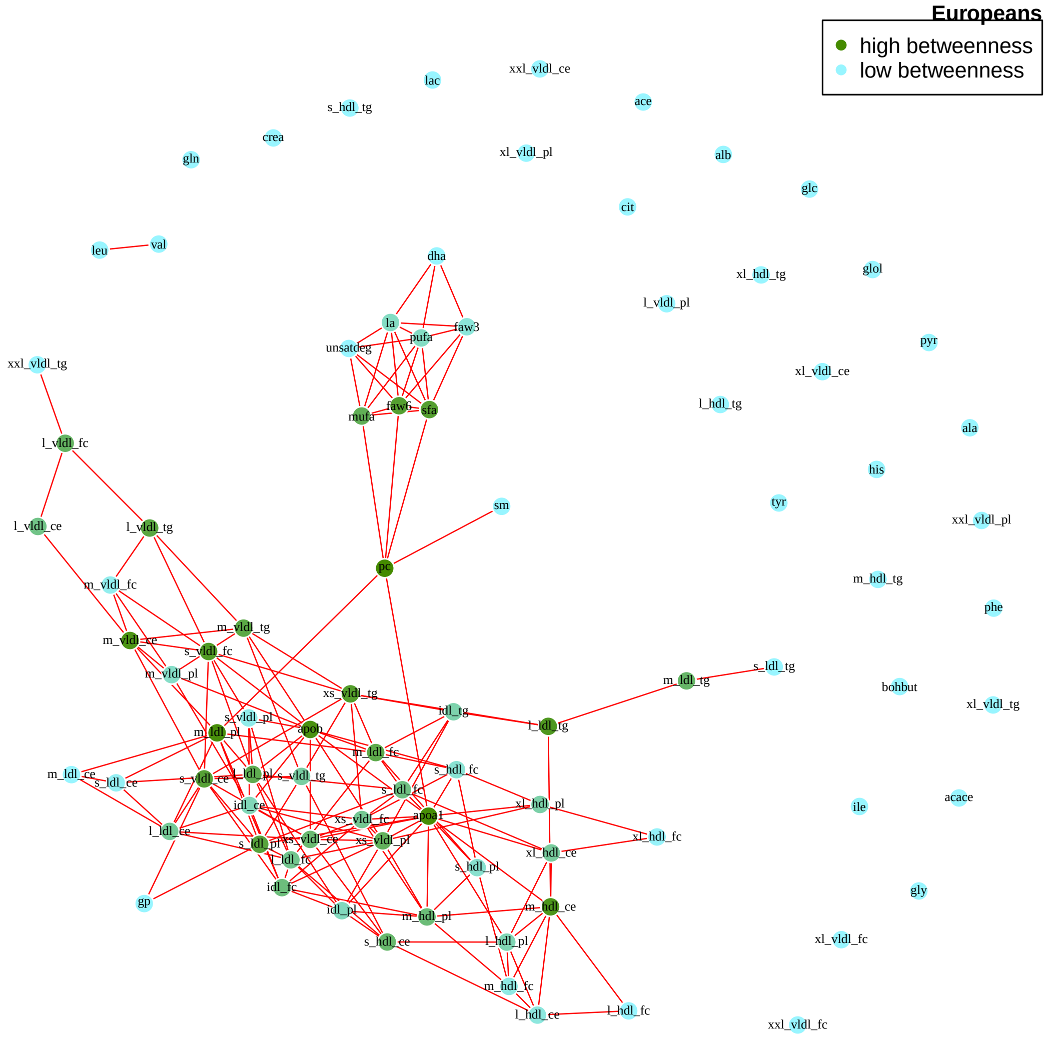

Figure 10. Individual network for the European ethnicity at baseline obtained applying the *AND* rule. An edge between two nodes is included in the graph if its posterior probability of inclusion is higher than 0.8. The colours of the nodes represent high/low levels of betweenness, indicating the degree of connectivity of the nodes within the graph.

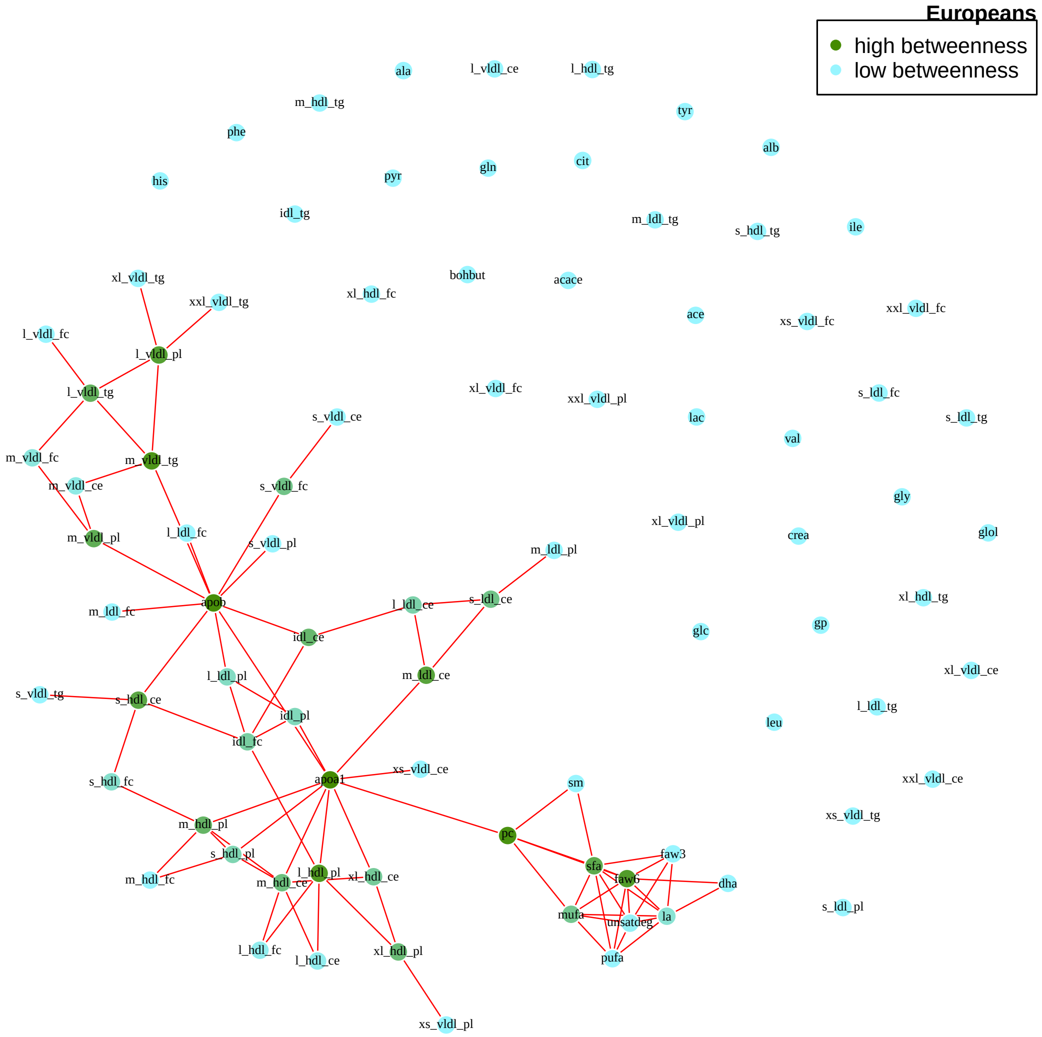

Figure 11. Individual network for the European ethnicity at follow-up obtained applying the *AND* rule. An edge between two nodes is included in the graph if its posterior probability of inclusion is higher than 0.8. The colours of the nodes represent high/low levels of betweenness, indicating the degree of connectivity of the nodes within the graph.

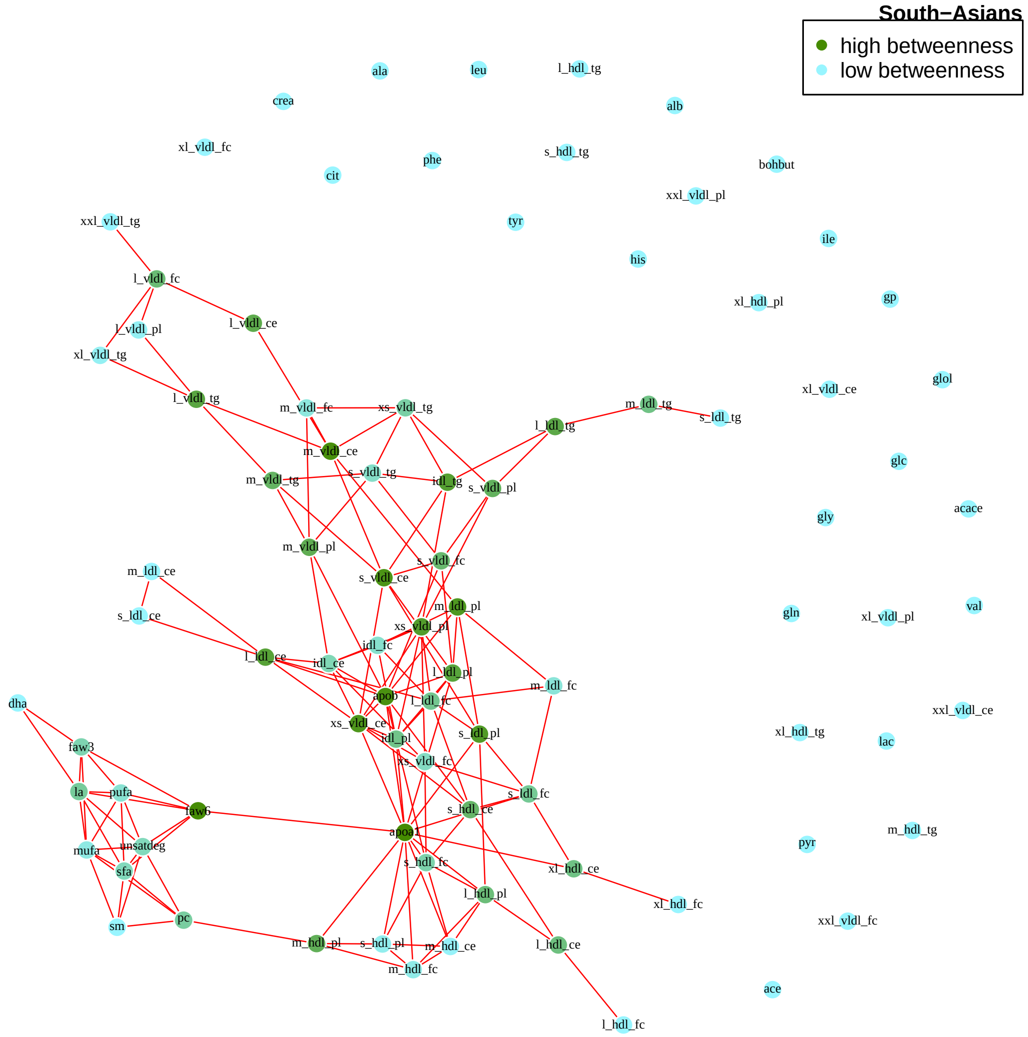

Figure 12. Individual network for the South-Asian ethnicity at baseline obtained applying the *AND* rule. An edge between two nodes is included in the graph if its posterior probability of inclusion is higher than 0.8. The colours of the nodes represent high/low levels of betweenness, indicating the degree of connectivity of the nodes within the graph.

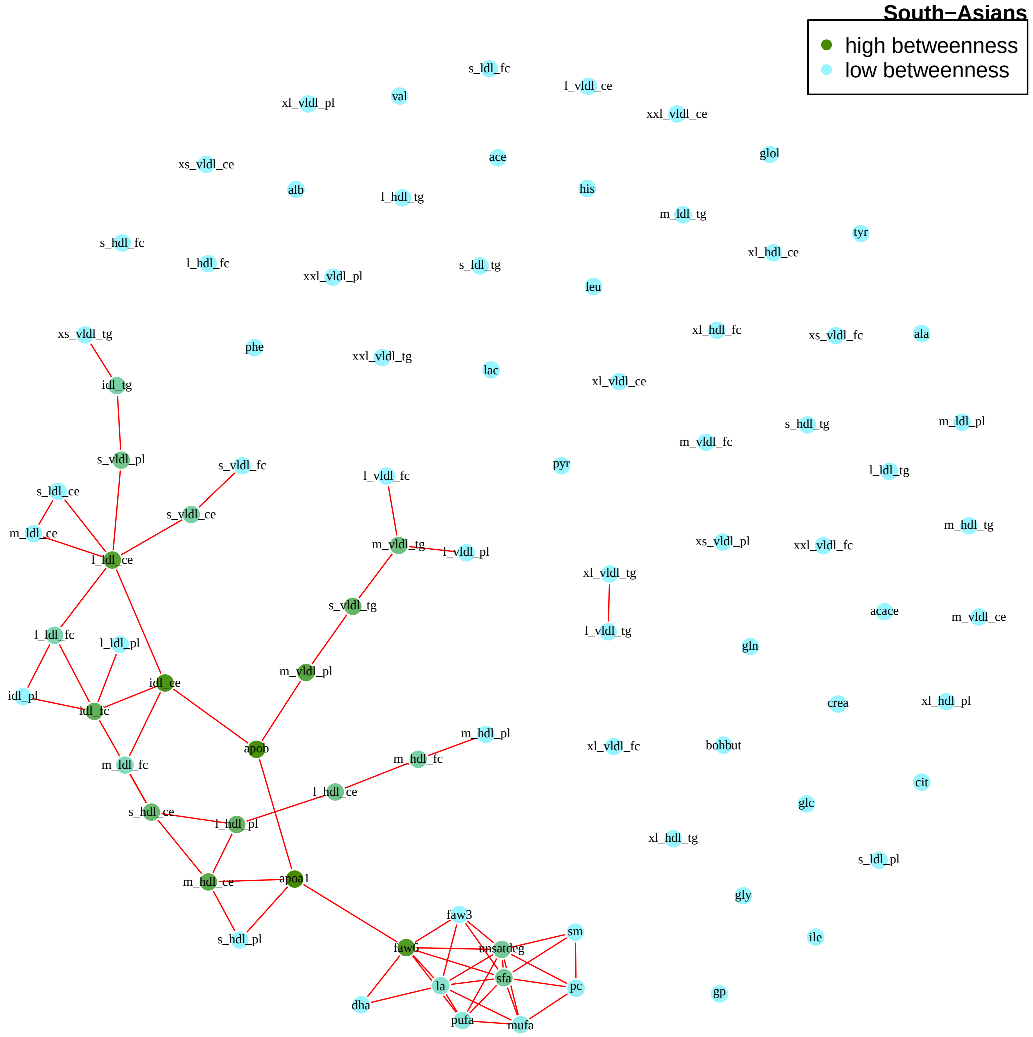

Figure 13. Individual network for the South-Asian ethnicity at follow-up obtained applying the *AND* rule. An edge between two nodes is included in the graph if its posterior probability of inclusion is higher than 0.8. The colours of the nodes represent high/low levels of betweenness, indicating the degree of connectivity of the nodes within the graph.

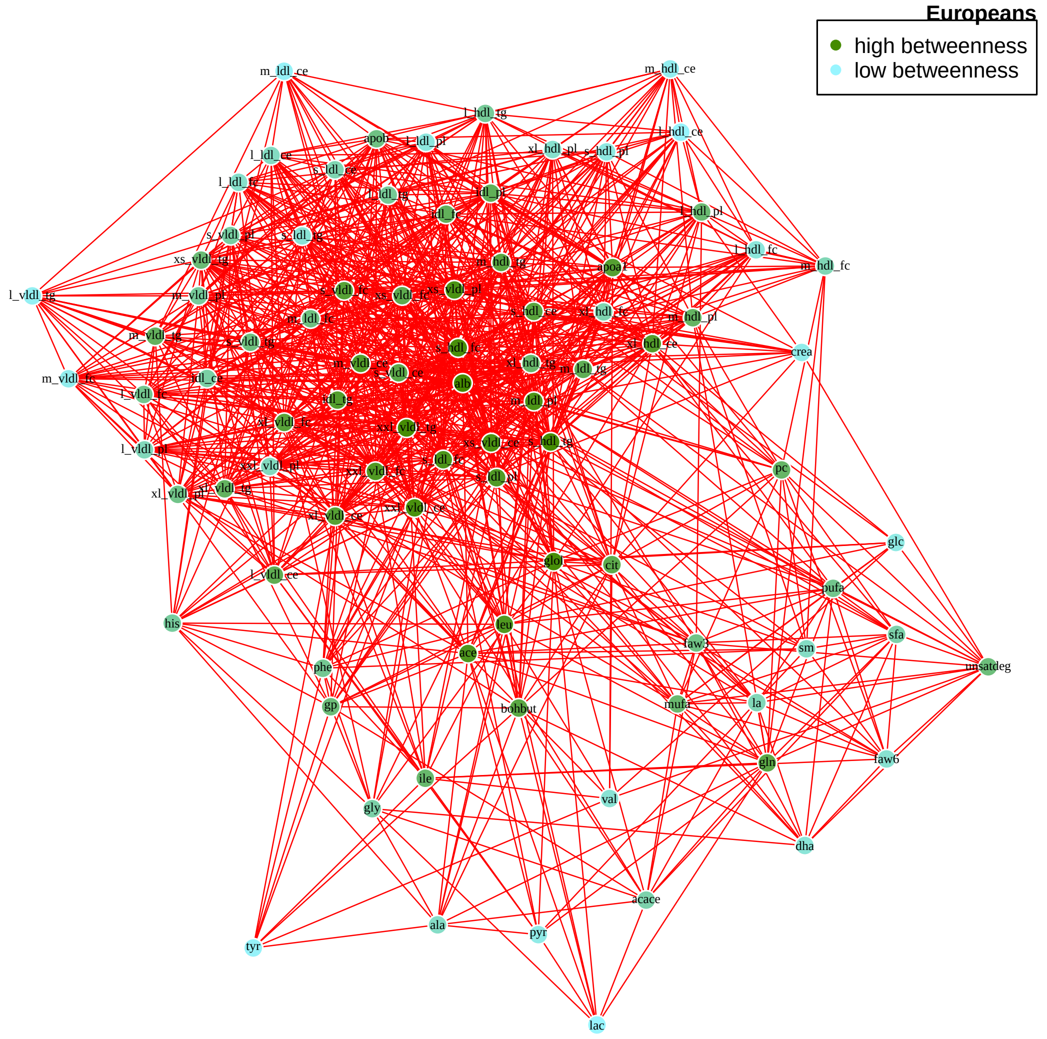

Figure 14. Individual network for the European ethnicity at baseline obtained applying the *OR* rule. An edge between two nodes is included in the graph if its posterior probability of inclusion is higher than 0.8. The colours of the nodes represent high/low levels of betweenness, indicating the degree of connectivity of the nodes within the graph.

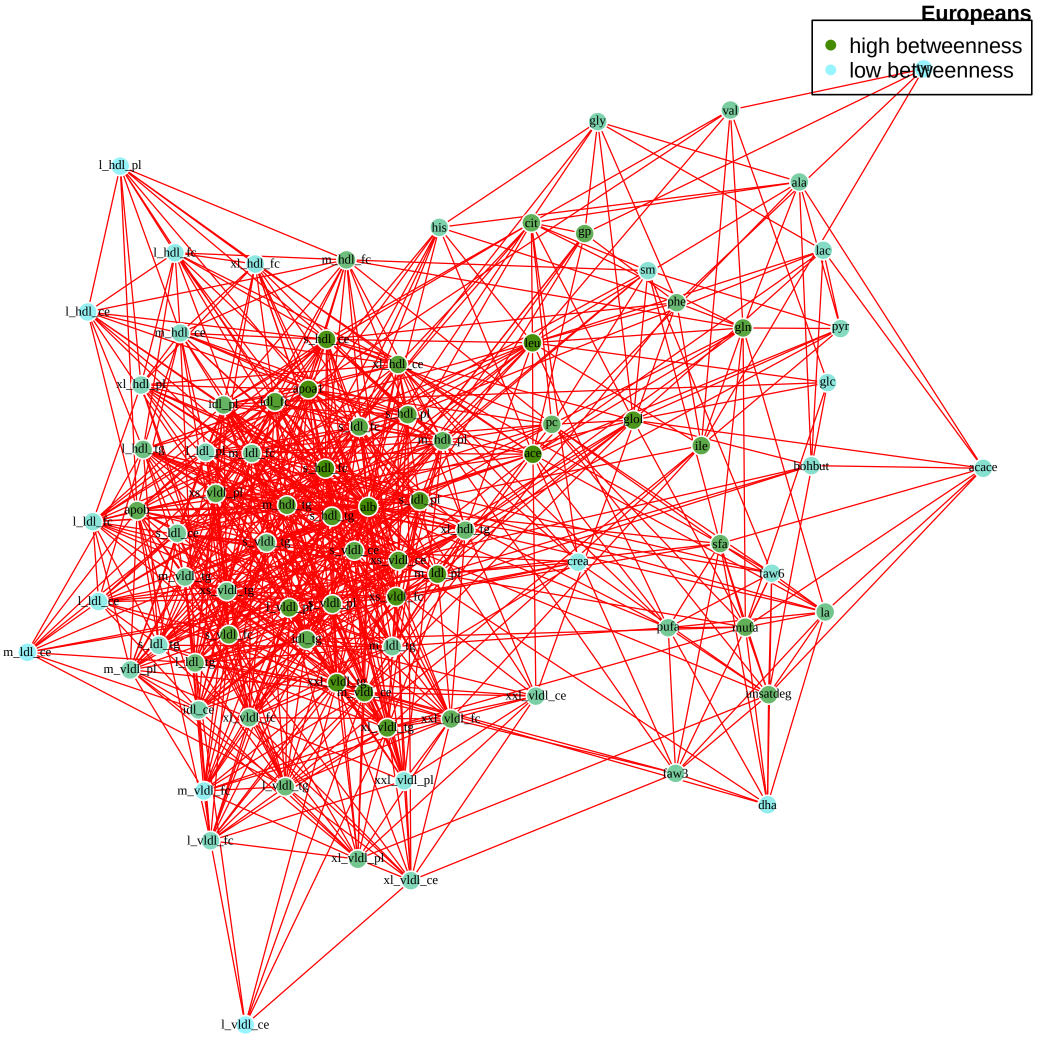

Figure 15. Individual network for the European ethnicity at follow-up obtained applying the *OR* rule. An edge between two nodes is included in the graph if its posterior probability of inclusion is higher than 0.8. The colours of the nodes represent high/low levels of betweenness, indicating the degree of connectivity of the nodes within the graph.

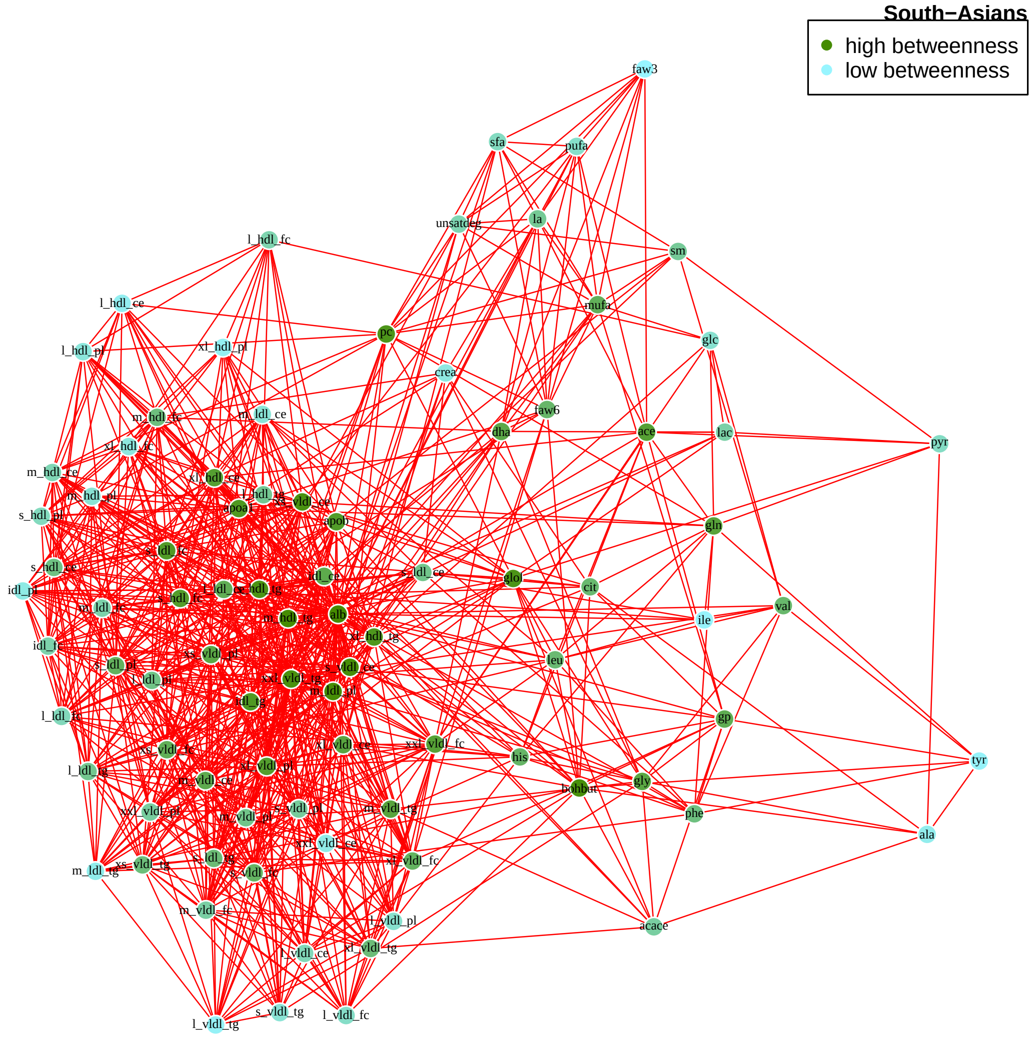

Figure 16. Individual network for the South-Asian ethnicity at baseline obtained applying the *OR* rule. An edge between two nodes is included in the graph if its posterior probability of inclusion is higher than 0.8. The colours of the nodes represent high/low levels of betweenness, indicating the degree of connectivity of the nodes within the graph.

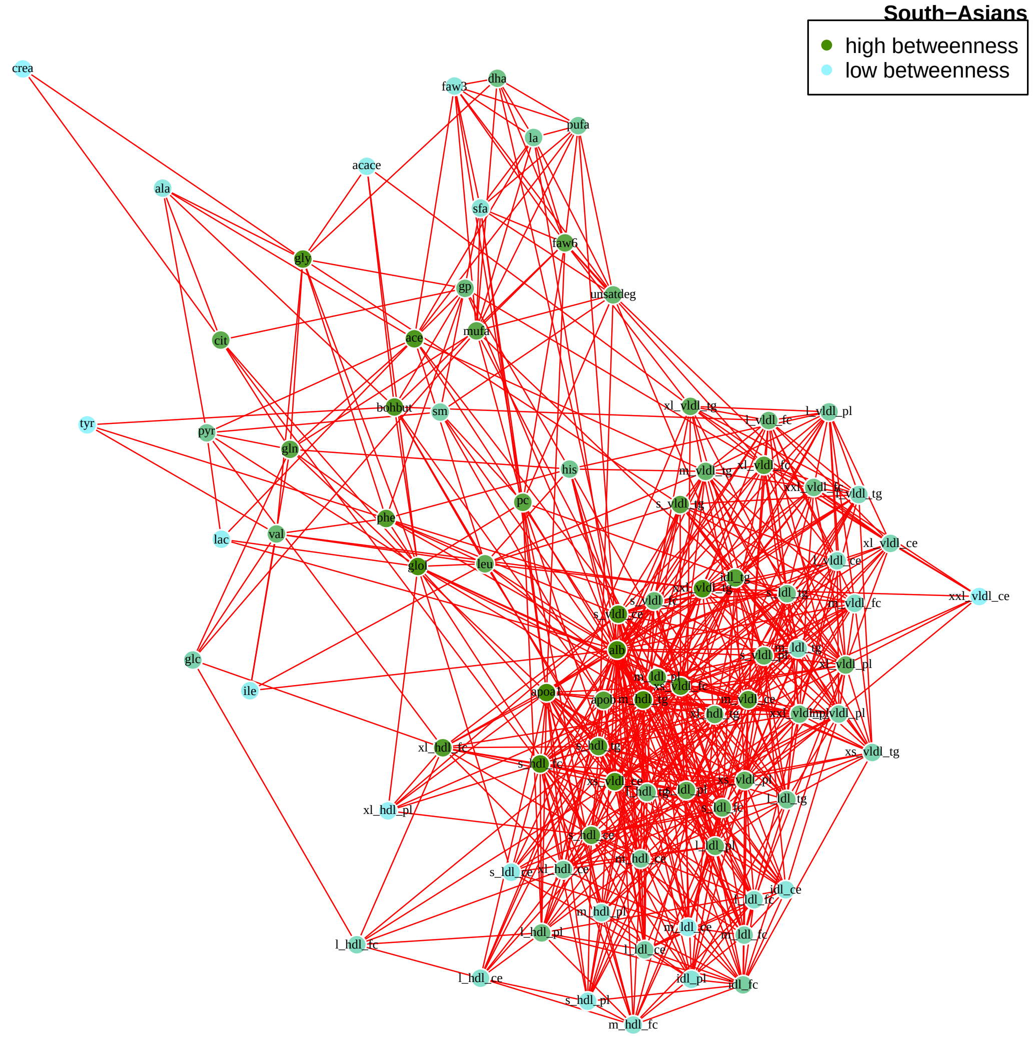

Figure 17. Individual network for the South-Asian ethnicity at follow-up obtained applying the *OR* rule. An edge between two nodes is included in the graph if its posterior probability of inclusion is higher than 0.8. The colours of the nodes represent high/low levels of betweenness, indicating the degree of connectivity of the nodes within the graph.

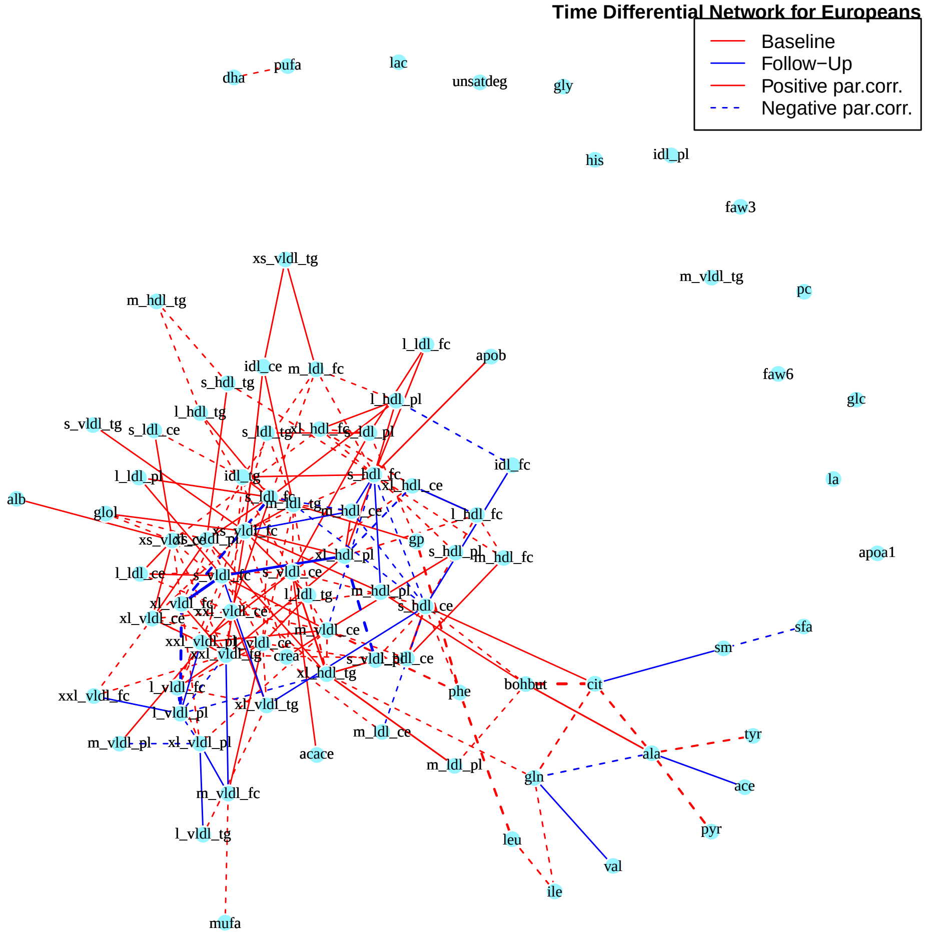

Figure 18. European Differential network between baseline and follow-up obtained with the *OR* rule. Red lines correspond to edges only present in the baseline network, while blue lines to those only present in the follow-up network. Continuous lines represent differential positive partial correlations, while dashed lines indicate negative ones.

Table 3. Anthropometric covariates, diabetes indicator, CVD indicators and other control variables. Every variable is used at time 1 (T_1) and time 2 (T_2).

Variable name	Label
Age	Respondent's age
WHR	Waist to Hip Ratio
Systolic blood pressure	Systolic blood pressure
Homa IR	Homeostasis model assessment Insulin Resistance
Sex female	Dummy variable for female sex (male as reference category)
Smoke_Ex	Indicator variable for ex smoker category
Smoke_Current	Indicator variable current smoker category
Pt_alcohol	Weekly quantity of alcohol consumed
Phys_score	Physical activity score
Edu_years	Years of education (assumed constant over time)
bl_hdl	Concentration of HDL
bl_trig	Concentration of blood Triglycerides
bl_chol	Concentration of blood Cholesterol
CHD	Indicator variable for the presence of Coronary Heart Disease up to T_1 and T_2
stroke	Indicator variable for the presence of stroke up to T_1 and T_2
Diabetes	Indicator variable for the presence of Diabetes Mellitus (type 2) up to T_1 and T_2
dm_treat	Indicator variable for the presence of drug treatment for diabetes up to T_1 and T_2
bp_treat	Indicator variable for the presence of blood pressure lowering drugs treatment up to T_1 and T_2
lipids_treat	Indicator variable for the presence of blood lipids lowering drugs treatment up to T_1 and T_2

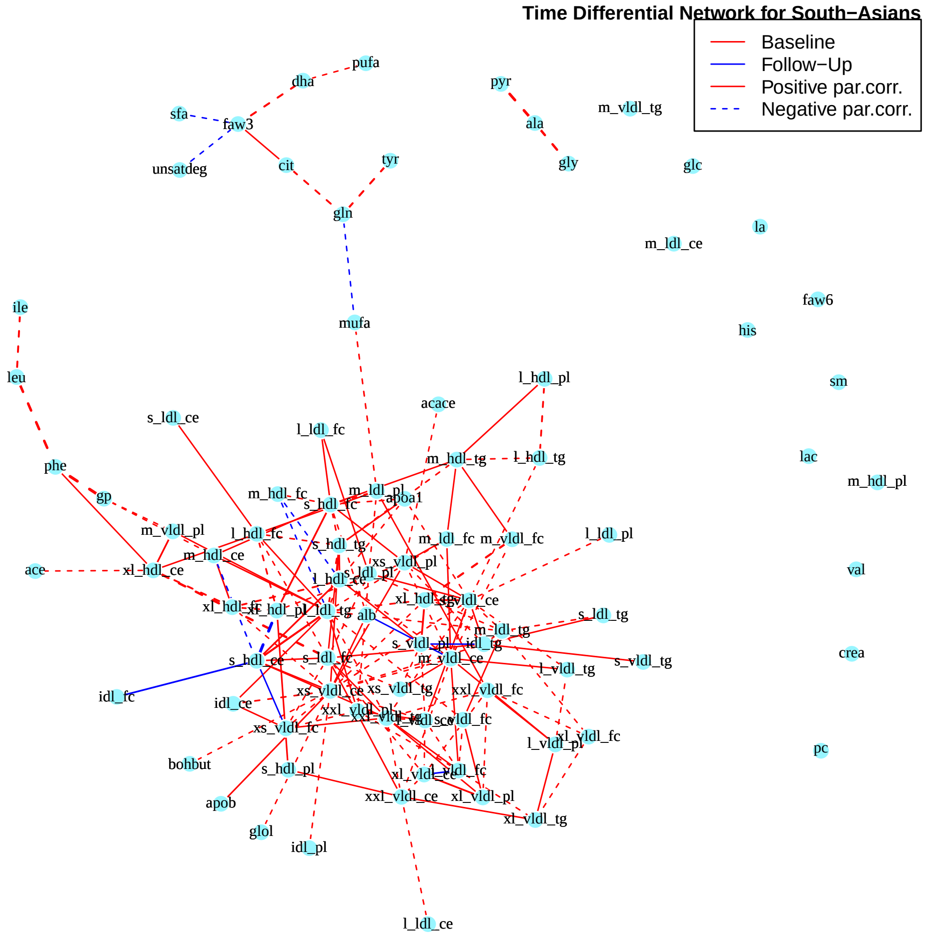

Figure 19. South-Asians Differential network between baseline and the follow-up obtained with the *OR* rule. Red lines correspond to edges only present in the baseline network, while blue lines to those only present in the follow-up network. Continuous lines represent differential positive partial correlations, while dashed lines indicate negative ones.

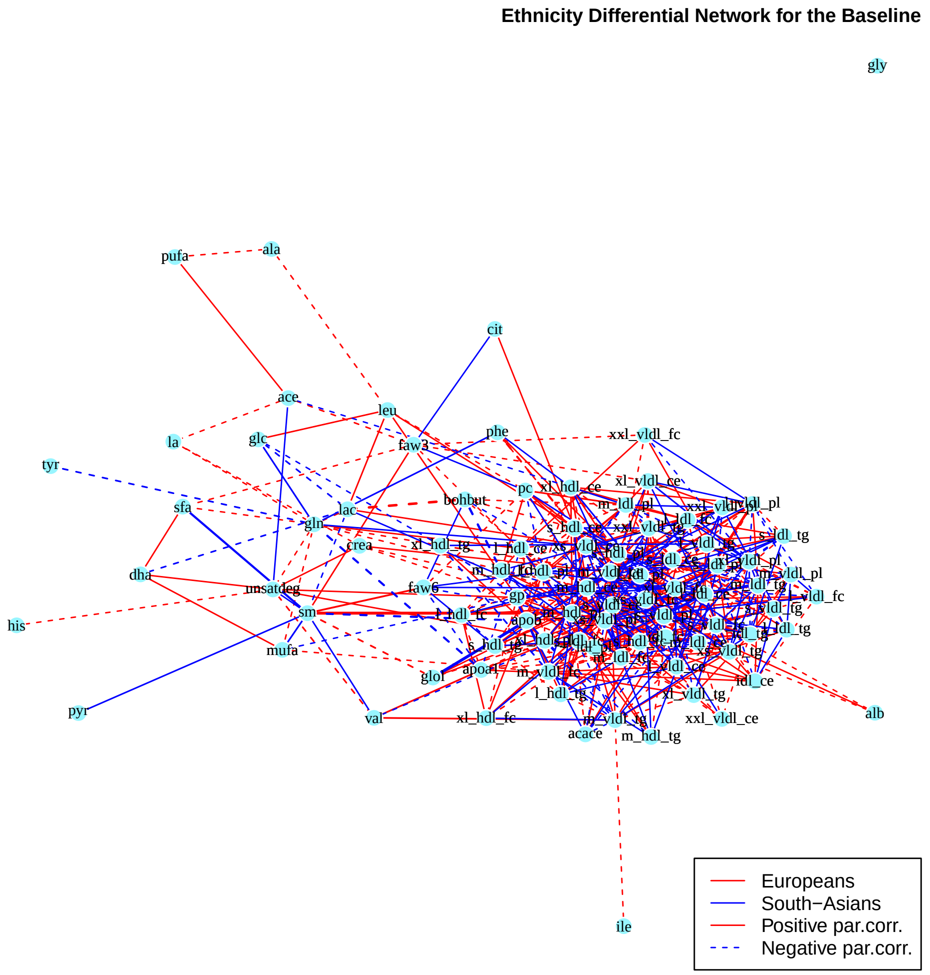

Figure 20. Differential network between Europeans and South-Asians at baseline obtained with the *OR* rule. Red lines correspond to edges only present in the European network, while blue lines to those only present in the South-Asian network. Continuous lines represent differential positive partial correlations, while dashed lines indicate negative ones.

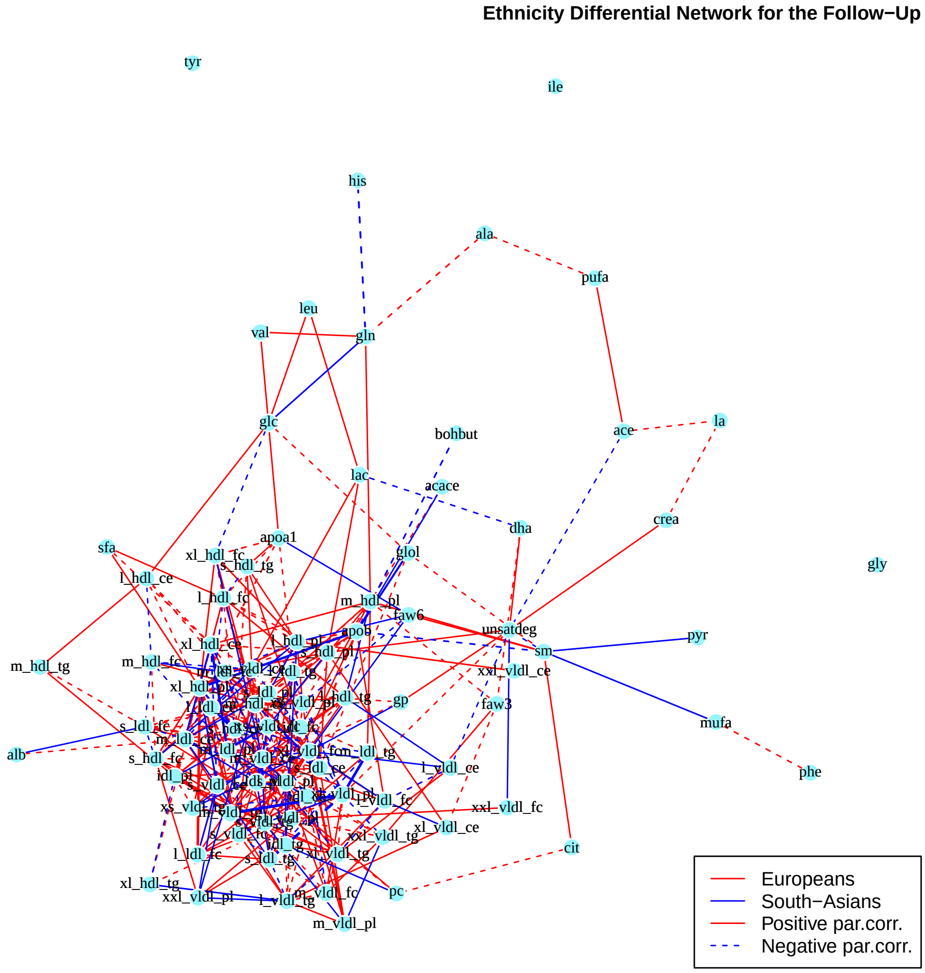

Figure 21. Differential network between Europeans and South-Asians at follow-up obtained with the *OR* rule. Red lines correspond to edges only present in the European network, while blue lines to those only present in the South-Asian network. Continuous lines represent differential positive partial correlations, while dashed lines indicate negative ones.

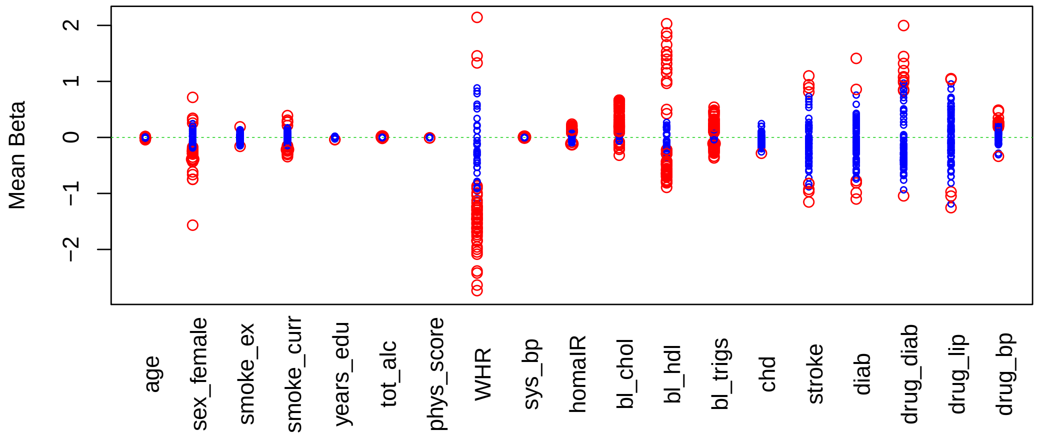

Figure 22. Posterior means of η_{lj} for the Europeans at baseline. Each dot represents the mean of the posterior distribution of a coefficient η_{lj}, $l = 1 \ldots, M$. Red dots denote coefficients whose 95% credible interval does not contain the zero.

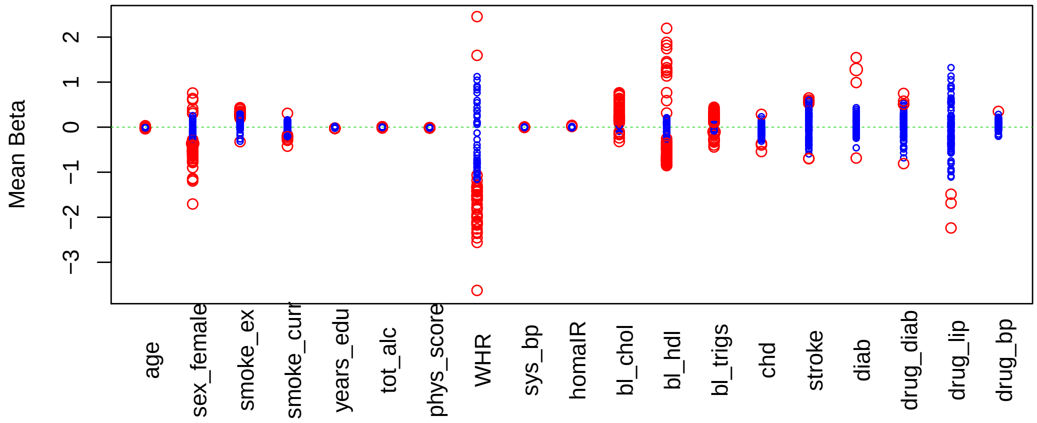

Figure 23. Posterior means of η_{lj} for the South-Asians at baseline. Each dot represents the mean of the posterior distribution of a coefficient η_{lj}, $l = 1 \ldots, M$. Red dots denote coefficients whose 95% credible interval does not contain the zero.

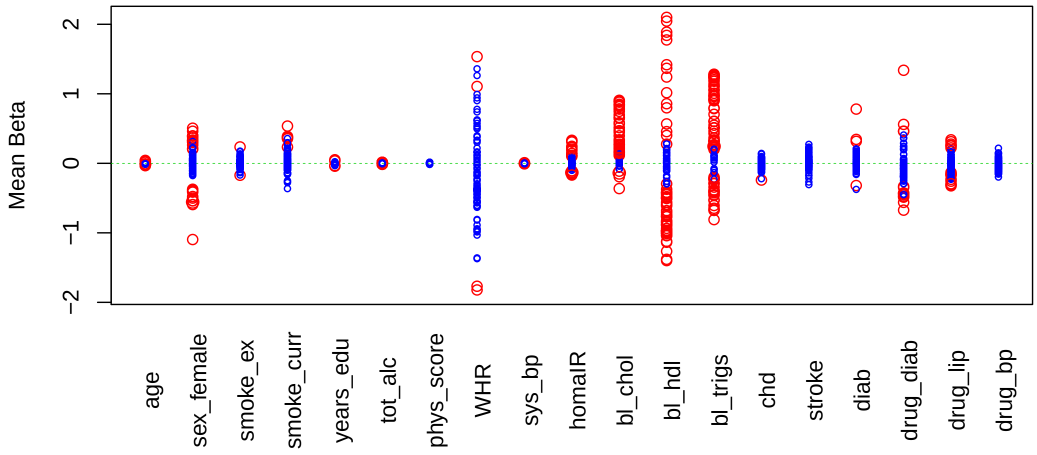

Figure 24. Posterior means of η_{lj} for the Europeans at follow-up. Each dot represents the mean of the posterior distribution of a coefficient η_{lj}, $l = 1\ldots, M$. Red dots denote coefficients whose 95% credible interval does not contain the zero.

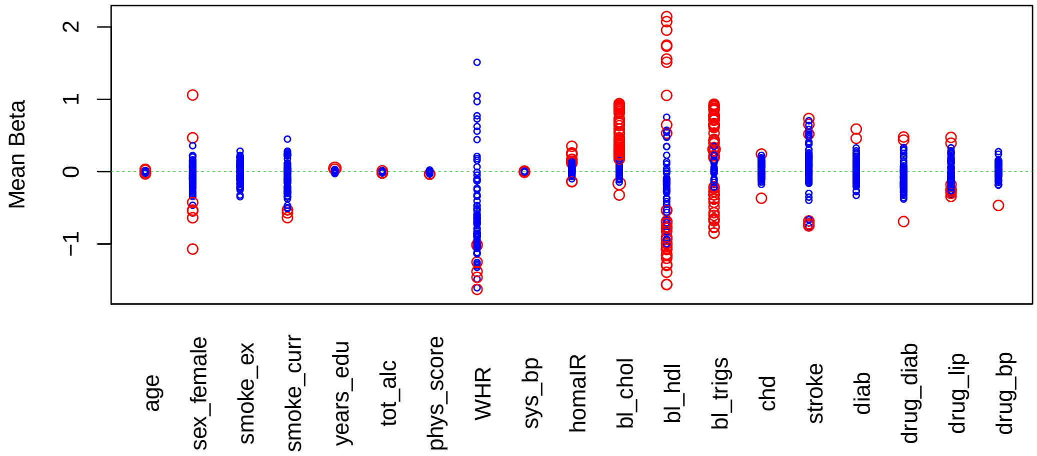

Figure 25. Posterior means of η_{lj} for the South-Asians at follow-up. Each dot represents the mean of the posterior distribution of a coefficient η_{lj}, $l = 1\ldots, M$. Red dots denote coefficients whose 95% credible interval does not contain the zero.
